# Support to address barriers to learning for learners who are deaf

**DOI:** 10.4102/ajod.v7i0.381

**Published:** 2018-10-22

**Authors:** Peter Mapepa, Meahabo D. Magano

**Affiliations:** 1 Department of Education, University of Johannesburg, South Africa; 2 Department of Psychology of Education, University of South Africa, South Africa

## Abstract

**Background:**

There is great importance in support services for successfully addressing the barriers to learning optimally or learners who are deaf. The study, though conducted in South Africa, has national and international appeal.

**Objectives:**

The aim of the study was to identify educator reflections on support services needed for them to address barriers to learning of learners who are deaf.

**Method:**

The study used a qualitative design for collecting data in natural settings. A sample size of 11 educators of learners who are deaf was purposively selected from two provinces of South Africa. The study used an open ended individual interview questionnaire.

**Results:**

Data was analysed using qualitative content analysis considering the context of the schools in which the study was carried out. Results showed that there was: limited curriculum support in special schools; lack of support and inadequate teaching and learning materials; overcrowding in one school and; limited support of multidisciplinary professionals in most schools.

**Conclusion:**

The study provided a framework for support services important for research, policy and practice. Of significance was the relevance of the Universal Design for Learning (UDL) theoretical framework in implementing support services programmes in schools.

## Introduction

Support services are needed in addressing barriers to learning of learners who are deaf and learners who are hard of hearing. These include the provision of appropriate teaching materials. Visual materials appropriate for learners who are deaf include pictures, diagrams and illustrations to enhance their understanding. It also includes slides and DVDs.

Availability of adults who are deaf is important for role modelling because this forms a link between the teacher with normal hearing and the learner who is deaf (Hauser et al. 2010; Humphries et. al. 2013). Adults who are deaf play an important role in sign language training and ensuring that the new learner who is deaf is introduced to deaf culture (Mcllroy 2010). The challenge facing most teachers today is that they have not been taught how to cope with diversity of learners now entering schools (Storbeck 2009:356).

Differentiation is important to set up individualised education plans (IEPs) to assist learners experiencing barriers to learning. Assessments are also adapted to consider the specific barriers of each learner. Storbeck (2011:354) maintains that most teachers in most developing countries are unable to differentiate, adapt material and use sign language. The Department of Education (DoE 1998:17) is concerned that assessment remains the responsibility of the specialist teacher. The specialist teacher is responsible for differentiating and adapting common examinations which is time-consuming. The DoE (2005) supports provision that is brought to the child rather than the child to the provision. For example, nutritional support is provided through the Schools Nutrition Programme and Health Promotion. Social support includes the provision of safe and clean water and the availability of proper sanitation (Gascon-Ramos 2008:63). Gason-Ramos (2008:64) is convinced that both psychosocial and material supports are important. The Department of Basic Education (DBE 2011) supports making provision for both curriculum and co-curricular support services which are important for the education of the learner who is deaf.

## Theoretical framework

The study is informed by the Universal Design for Learning (UDL) which seeks to make expert learners of all students (Teaching Excellence in Adult Literacy [TEAL] 2010:2). The UDL is a theoretical framework that focuses on assisting teachers to plan learning with intent to meet the diverse needs of learners (Rose et al. 2016). The theory utilises multiple approaches to engage learners in the learning process (Rose, Meyer & Hitchcock 2005). The UDL sets out goals and uses tools, materials and assessments geared to address the different barriers to learning (Rose & Meyer 2006). Teaching Excellence in Adult Literacy (2010:2) maintains that learners are heterogeneous in ability and that the best way to educate them is to use different kinds of support. This also involves scaffolds teaching learners starting with the easier, more basic concepts, advancing gradually to the more advanced abstract concepts.

Learners have different kinds of abilities. Yet, the general curriculum treats them as a homogenous group. The UDL is an important lens for the study on learners who are deaf to gain the necessary learner support. Rose and Vue (2010) support a curriculum that is flexible and effective for every learner whether average or slow. Ralabate (2011) maintains that a curriculum that is responsive to learner diversity reduces barriers to instruction. Emphasis of early special education legislation was on the need for learners to be taught in the least restrictive environment (Marshark & Spencer 2003; Rose & Meyer 2006). The UDL standards emphasise teaching learners who are deaf using simplified graphical information. Rose, Meyer and Hitchcock (2005) are of the view that IEPs are effective methods to meet the needs of every learner. Teaching methods have to be flexible and adjusted through consistent monitoring of student progress (Rose et al. 2016). Learners who are deaf learn effectively if a variety of materials are available to support their learning. With UDL, various methods and materials are used to assess student knowledge, and skills, making sure that assessments are valid. Rose and Vue (2010) maintain that UDL motivates learners to be self-directed active learners using visual materials which they can understand and interpret. Learners who are deaf can also assess their own learning needs, monitor their progress and remain motivated in their learning (Rose &Vue 2010).

## Rationale

Various forms of support are needed for learners who need additional support, such as the learner who is deaf. The Department of Education (DBE 2014) suggests that support of learners who are deaf is important for them to learn effectively. It recommends having a South African Sign Language (SASL) interpreter to assist the classroom teacher who is not competent enough in SASL. It is also important to have an educator who has been trained in SASL to provide teaching. Psychosocial support to manage behaviour challenges associated with the child’s disability is also necessary (Gascon-Ramos 2008:64). The DBE (2014:16) suggests support needed such as assistive devices, specialised equipment and teaching and learning support materials. On curriculum design and implementation, it is important for curriculum differentiation and emphasis on IEPs to meet the individual needs of learners (DBE 2014:16).

Learners further need to be trained in areas of language development and to have good mentors who provide the necessary guidance (Storbeck 2000:58). In the need for support for learners who are deaf, research studies in the United States have indicated that using multimedia promotes effective learning for the learners (Marshark & Hauser 2013). The need for specialist support staff is one of the greatest support needs (Storbeck 2009:356). Assistive devices such as hearing aids can be very useful for some learners. Visual materials and pictures, DVDs and videos help learners understand better what they are learning (Storbeck 2009:360). Curriculum differentiation helps to motivate learners through IEPs and adapted assessments, yet it is a challenge in most developing countries (Storbeck 2009:356).

However, governments globally have managed to change attitudes of their citizens towards people who are deaf from negative to positive (Hauser et al. 2010:487). Policies and legislation are some of the available support systems, for example, support for inclusion and education for all (EFA). Education for all is about making it possible for all learners, despite their barriers to learning, to be able to access the curriculum. The government of South Africa used the Education White Paper 6 to build an inclusive education and training system (Department of Basic Education [DBE] 2011). This was followed up by the Screening Identification, Assessment and Support (SIAS) document which clarified issues mentioned in the White Paper 6 for action and implementation (DBE 2011, 2014).

Support should be brought to the child rather than expecting the child to play an active role in seeking support (Marshark & Hauser 2013; Rose & Meyer 2002). The DBE (2011) stresses that support services must protect and ensure safety of learners. As part of social support, the social welfare services play an active role in learner support (Gascon-Ramos 2008). The DBE (2011) stresses the importance of maintaining balance between curriculum and co-curricular supports for learners who are deaf. Curriculum support is a whole school issue. Curriculum is what is taught, how it is taught and under what conditions it is taught (DBE 2011). For curriculum to be effectively implemented, it has to be flexible and accessible to all learners including the learners who are deaf (Fernandes & Myers 2010).

The educator provides some learners’ curricular and co-curricular support needs (Storbeck 2009). The teacher has a responsibility to motivate learners by using appropriate teaching strategies and active learning techniques. This brings intrinsic motivation, eagerness and purpose to learn through self-directed tasks (TEAL 2010:2). Intrinsic motivation is important for self-knowledge and self-control in the learning situation, avoiding attribution and uplifting the value of educational tasks (Gascon-Ramos 2008:59). It is important for learners to know who they are, what they can do and what they want to achieve. It also helps learners who are deaf to access the wider society. This is achieved by getting motivated to read and write in English as a second language, for example. Personalising education helps create positive involvement in activities that have personal meanings to learners (Gascon-Ramos 2008:63). Gascon-Ramos (2008:59) explicates the positive benefits of involving parents in effective curriculum delivery of learners who are deaf. Parental involvement is part of providing access and motivating learners to want to learn (Solvang & Haualand 2014).Additional support is important and can be provided by the district-based support team. The DBE (2011) asserts that some of the support services facilitated by the school-based support teams (SBSTs) include staffing, assistive devices, assistance in curriculum differentiation, whole school development, School Management Team (SMT) training, educator development, parental development and specialised facilities.

Curriculum support is a whole school issue. Curriculum is what is taught, how it is taught and under what conditions it is taught (DBE 2010). For curriculum to be effectively implemented, it has to be flexible and accessible to all learners including learners who are deaf. The following research question was formulated to guide the study: ‘What are educator reflections on support services needed for them to address barriers to learning of learners who are deaf?’

## Research design

The study is a qualitative design in data collection and analysis and interpretation of data (Creswell 2007). The study used an interpretive design paradigm with the intention of obtaining teachers’ perspectives on what they said on their perceptions on the available support services to enable teachers to address learners’ barriers to learning. Qualitative methods consider several realities based on the person perceiving it and the context on which it is perceived (Guba & Lincoln 2005). The qualitative method enabled variables to be observed in natural settings (Denzin & Lincoln 2000; Smith, Flowers & Larkin 2009).

The use of qualitative methods enabled individual participants to give different experiences and perceptions. The study followed a phenomenological approach allowing individuals to express their lived experiences (Babbie & Mouton 2011:28; Joubish et al. 2014).

In the qualitative study, a total of 11 primary school educators of learners who were deaf were purposively selected to participate in the interviews. Participants were all teachers of learners with deafness, thus providing the same criteria (Onweuegbuzie & Leech 2007). Participants were drawn from two provinces at special schools for learners who are deaf, teaching Grade One to Seven. Both sexes were interviewed, consisting of eight women and three men. Eight participants disclosed their ages. The age range was from 26 years to 60 years with four of them in the age range of 51–55 years. Three of the educators did not want to disclose their ages.

### The instruments

The interview elicits information from the interviewee (McMillan & Schumacher 2010:205). An interview involves a small number of people being interviewed on their perspectives on the programme or situation at stake (Creswell 2007). The interview schedule had two sections. The first section sought information on biographical data. The second section was on academic wellness with reference to adaptation of the curriculum and support services in academic and communication with emphasis on issues of adaptation. The interview schedule instructions were simple, clear and concise as recommended by Guba & Lincoln (2005).

### Data analysis

Data analysis involved organising and synthesising the data (Babbie & Mouton 2011:388). Analysis helped in ensuring that a correct record of the interviews was reflected in the analysis (Guba & Lincoln 2005). Coding was done by thoroughly analysing and selecting important elements in the interviews (Babbie& Mouton 2011:492). Coding helped to categorise concepts and identify significant parts of the data (Creswell 2007).

Patterns were developed in the categories, grouping together codes with similar meaning which were further developed as themes, which can be likened to core categories (Babbie & Mouton 2011:501).

## Ethical consideration

Ethics Clearance was provided by the Research Ethics Committee of the University of South Africa dated 17 November 2014, reference number 2014NOVEMBER/06849962/MC.

## Findings

The findings led to the following four themes:

- Curriculum adaptation is an important support system.
- Lack of support in appropriate teaching or learning materials.
- Overcrowded learning space in one school.
- Limited support of multidisciplinary professionals in most schools.

### Curriculum adaptation is an important support system

Participants in the qualitative study indicated their perspectives on professional support in curriculum. Participants reported that the current state of the curriculum adaptation in special schools was limited. This is clear from comments by some of the participants.

Participant D commented:

‘Curriculum adaptation by the Department of Education is minimal.’

This comment suggests that curriculum adaptation was only available to a small extent. Participant C noted that the DoE was doing little to adapt the Curriculum Assessment Policy Statements (CAPS) curriculum.

Interviewee C commented:

‘The Provincial Education Department expect from us to offer Caps curriculum without adaptation.’

Normally, when a new curriculum is introduced, teachers are trained in the teaching of the curriculum. For the CAPS curriculum, this was done for all subjects and phases. Training was done for the foundation phase, and then it was extended to the intermediate and senior phases. Training of teachers involved all the teachers including teachers of the hearing-impaired. Comment by Interviewee C suggests that the training did not consider disabilities such as hearing impairment.

Participants E and A gave comments that support those given by C as can be seen below.

Participant E commented:

‘Especially subject facilitators do not understand the needs of deaf learners.’

The subject facilitators are the professionals based at regional officers who are responsible for training of teachers and ensuring effective implementation. The comment suggests no notable effort by the curriculum implementers to offer adaptation alternatives to teachers of learners who are deaf.

Participant A commented:

‘There is need for more support from inclusive education sector on implementation of the curriculum.’

The inclusive section of the DoE is responsible for ensuring that learners experiencing barriers to learning are well supported in their learning.

Participants G suggested that the DoE was not adapting examinations addressing specific needs of learners with hearing impairment.

Participant G commented:

‘Deaf friendly examinations instead of expecting teachers to adapt.’

With adequate training, teachers should be able to adapt the examinations. However, as an answer to the follow-up question, the participant clarified the view that it was a responsibility of the teachers to adapt as seen in the following extract:

‘Who do you expect to adapt deaf friendly examinations?’ (Researcher)

‘[*Looking surprised by the question*] The Department of Education should adapt not us the teachers.’ (Participant G)

‘How will that help the learner writing the examination?’ (Researcher)

‘[*Confidently*] It helps the learner to personally read the questions in the examinations. It also helps because it saves time.’ (Participant G)

Participant A commented:

‘There is need for more support from inclusive education sector on implementation of the curriculum.’

Inclusive section of the DoE is responsible to ensure support for learners experiencing barriers to learning.

Participant G suggested that the DoE was not adapting examinations addressing specific needs of learners with hearing impairment.

Participant G commented:

‘Deaf friendly examinations instead of expecting teachers to adapt.’

With adequate training, teachers should be able to adapt the examinations. However, as a follow-up question the participant clarified the view as seen in the following interview extract:

‘Who do you expect to adapt deaf friendly examinations?’ (Researcher)

‘[*Looking surprised by the question*] The Department of Education should adapt not us the teachers.’ (Participant G)

‘How will that help the learner writing the examination?’ (Researcher)

‘[*Confidently*] It helps the learner to personally read the questions in the examinations. It saves time and ensures that the questions are interpreted they were intended by the examiner.’ (Participant G)

From the above extract, participants feel that the DoE lacked assessment policy to adapt examinations before they were dispatched to special schools.

### Lack of support in appropriate teaching or learning materials

Support in adaptation is also viewed in terms of appropriate teaching or learning materials.

Participant E noted that:

‘The Department of Education should find ways of procuring appropriate teaching and learning materials for the learners.’

This sentiment from the participant points to a need for the procurement on the shoulders of the DoE.

Participant G also suggested the need for:

‘Provision of teaching and learning materials to improve academic work.’

These sentiments suggest that the DoE was not playing an active role in effective learning of learners with hearing impairment because it was not assisting schools with the appropriate teaching or learning materials.

However, notable support was evident in the procurement of hearing aids to assist some learners who are hard of hearing.

Interviewee C noted that:

‘support comes in provision of hearing aids by health personnel.’

This suggests that the government through the Department of Health was playing a positive role in providing hearing aids. Hearing aids are a positive support to enhance their learning.

Provision of visual materials to enhance learning of learners who are deaf is an important support lacking in schools.

Participant H felt that:

‘It is easier, more natural for deaf learners to translate the visual SASL into meaning and knowledge.’

From the participant’s words, it is clear that communication was made effective enough by visual materials accompanied by words to assist the learner who is deaf.

### Overcrowded learning space in one school

In one school, there was limited learning space leading to overcrowded learners in the school.

Participant J noted that:

‘More classrooms are needed for learners to learn in a free space.’

Participant J indicated that:

‘There is lack of facilities for skills training at our school. With inadequate facilities, teaching of skills is not effective enough.’

### Limited support of multidisciplinary professionals in most schools

Participants felt that there was limited multidisciplinary support in most schools for learners with hearing impairment. The following views by participants D, E, I and J reflect that participants expected a wide range of professionals to support teachers in school setting:

‘Support comes through SBST, SAPS, health personnel assist in addressing barriers to learning.’ (Participant D, female, 51 years, grade 2 teacher)

‘The school does not have many health professionals, for example, Counselors, Social Workers, Physiotherapists, Occupational Therapists and Dietician.’ (Participant E, female, 45 years, grade 6 teacher)

‘The health promoting schools by the Department of Health by nature is a good example of support.’ (Participant F, male, 54 years, grade 7 teacher)

‘The SBST invites SAPS members to speak about safety and other issues.’ (Participant I, female, 48 years, grade 1 teacher)

‘Multidisciplinary support is not complete.’ (Participant J, female, 35 years, grade 3 teacher)

Participants F and J’s comments show that in some schools, support of a multidisciplinary nature was not present or was inadequate. However, participants D, F and I appreciated the support given by some of the professionals from outside the school coming to support the school in a multidisciplinary approach. The schools’ SBSTs play an important role in facilitating the multidisciplinary approach. This was only possible where the SBST was active. However, if the SBST was not active enough, it would lead to the opposite happening. This is evident in a comment by one of the participants.

Participant J said:

‘SBST does not give support.’

And:

‘We need interpreters.’

The participant’s comment on interpreters is important for educators who were not conversant in SASL.

Participants noted that networking was limited. With limited networking, educators were not able to learn from each other’s experiences. This impacted negatively in how they addressed barriers to learning.

## Discussion

The findings from this study led to emerging new themes:

- the need for appropriate training of teachers,
- set up specialised material units,
- create enough learning space in rural schools, and
- create multidisciplinary teams in all schools.

### The need for appropriate training of teachers

Several support services are not well provided for. Findings from the qualitative study revealed that the current state of the curriculum adaptation in special schools was limited. A study by Storbeck (2000:58) in South Africa discovered that most teachers of the deaf failed to adapt because they had not trained in deaf education. Other studies endorse the same argument, for example, Glaser and Van Pletzen (2012) indicated that only 14% of teachers of learners who are deaf have well-developed SASL. DEAFSA (2009) reported of shortages of educators who are deaf to support the deaf education system. Lack of training also manifests itself when educators fail to identify barriers to learning. Most of the participants indicated that the DBE offered minimal and uncoordinated support for curriculum adaptation. Experience from the United States also yielded similar results. The California Department of Education (1999) found out the fact that uncoordinated programmes in schools and in regional set-ups were a hindrance to curriculum adaptation. Participants in this study felt that monitoring by subject facilitators was not effective enough because of limited awareness of the needs of learners who are deaf. The subject facilitators are the professionals based at regional officers who are responsible for training of teachers and ensuring there is effective implementation. Lack of effective support has a negative effect on the quality of education of learners with hearing impairment. A Swedish study by Haualand and Allen (2009) revealed that the level of education of learners with hearing impairment was poor, low and unacceptable. The Department of Education (2014) in South Africa supports the findings by acknowledging that there are low levels of support for learners with hearing impairment. Storbeck (2009:357) hinted that there is a need for well-planned psychosocial support to manage behaviour challenges associated with the child’s deafness. The Department of Education (2014:16) suggests that there should be specialist support staff. Storbeck (2009:359) discovered that the level of support for South African learners with hearing impairment is generally low.

Qualitative studies revealed that learners who are deaf acquire limited use of language (Storbeck 2000:58). Participants clearly voiced that learners who are deaf face several challenges mastering content subject material and engaging in an independent study. Prevalent language challenges with the language of teaching and learning also interfere with the ability to understand and comprehend concepts. Learners who are deaf have very limited vocabulary as a result of not being able to engage easily into incidental learning. The Department of Education (2005) observes that in a mainstream classroom situation, the learner who is deaf can be seen to exhibit difficulty in spelling and vocabulary. They also demonstrate difficulty in interpreting information and appear to tire or give up easily (Schirmer 2001:84; Scott 1996).

Participants also spoke negatively about the lack of deaf-friendly examinations. Teachers were expected to adapt the examinations so that they could be accessed by the learners who are deaf. The challenge with examinations is further compounded by the fact that some of the learners have additional challenges. In the United States, a third of learners who are deaf had additional disabilities (Gascon-Ramos 2008). It is the responsibility of the specialist teacher to differentiate and adapt common examinations. Low levels of support in assessment on examinations are a concern for many researchers and educators in deaf education (Storbeck 2009:354).

### Set up specialised materials unit

The unique findings of the study unearthed the lack of appropriate teaching learning materials. Other studies highlight the importance of visual materials (Storbeck 2000). Appropriate teaching and learning materials are necessary not only for group learning but also for IEPs (Gascon-Ramos 2008:64).

### Create enough learning space in rural schools

The challenge of facilities can be a challenge to the not so well resourced rural schools. Learners at such a school do not pay fees and are dependent on government grants for their survival. Insufficient learning space can make the implementation of IEPs challenging. A study by the Virginia Department of Education (2012) in the United States found out that small classes yielded better results for learners who are deaf in academic work, while large classes led to poor performance.

### Create multidisciplinary teams in all schools

Participants felt strongly about the need for a multidisciplinary approach to support teachers. Several studies emphasise the importance of the multidisciplinary team (Gascon-Ramos 2008:64; Johnson 2004; Reagen 2008). Professional support includes having professionals such as physiotherapists, occupational therapists, speech therapists, nurses, audiologists, counsellors, dieticians and social workers (Kirk, Gallagher & Anastasiow 1997:110).

School-based support team plays an active role in addressing some of the barriers.

However, some of the SBSTs were not active enough in this study. The Department of Education (2011) also emphasises the special role of the SBSTs in providing support for the education of learners experiencing barriers to learning including learners who are deaf.

Findings from the study revealed that there was low level networking among educators such that they could not learn from each other. Studies by Schirmer (2001:86) in the United States and Gosjean (2001) in Switzerland discovered that lack of networking led to teachers falling behind in developments in deaf education. This impacted negatively in how teachers addressed barriers to learning of their learners.

## Contribution of the study

The study contributes to the education of learners who are deaf in the following manner: The study contributes in terms of research, policy and practice. Underpinned in the study is theoretical significance and influence of the UDL. In South Africa and other African countries, inclusive education policies and practices are influenced by global trends. The study acknowledges the role of Dalton, Mckenzie and Kahonde (2012) in exploring ways to implement UDL with practical training of teachers and therapists. This study makes further propositions and directions on how further research can be carried out on UDL. The study provides a framework for support services in addressing barriers to learning of learners with hearing impairment. The researchers hope to share the findings with policy-makers in order to influence policy. This includes the need for provision of support in curriculum such as provision of appropriate teaching and learning materials. The researchers will also share the findings with educators of learners who are deaf to contribute to practice. Educators have a unique role because they influence the direction and extent in which the different support systems in the education of the learner are pointed towards.

## Limitation of the study and conclusion

The study is of a small scale based on two provinces with a track record of deaf education. Future studies can be carried out to involve more provinces and more participants. For example, only one educator indicated that the learning space was inadequate. Future studies can explore that assertion; this helps to see if schools have enough learning space for the learners or whether it was an isolated case for that particular school.

The study suggests that there is a need to enhance the extent of support in schools for learners who are deaf. Such support is minimal at the present moment. Furthermore, there is very little training currently available for teachers in sign language and curriculum adaptation. There is little meaningful support from DBE officials in terms of assessment with teachers taking a lot of time to adapt examinations. The DBE should play a more meaningful coordinating role in order to facilitate training, curriculum support and assessment of learners who are deaf. The provision of appropriately adapted teaching and learning materials would go a long way in ensuring barriers to learning of learners who are deaf are well addressed.

## Recommendations

As a contribution to the provision of learner support, this study recommends that adapted books and materials be availed to facilitate learning across all subjects. These books and materials would encourage visualisation. The DBE can play a leading role in this kind of support. This study recommends that there is a need for schools to have non-teaching professionals within the school setting to support social, emotional, career, spiritual and physical needs of learners. This study recommends that the DBE support to schools is important in organising training and provision of adapted teaching materials. In addition, different kinds of support are needed from parents, and governmental and non-governmental organisations to assist the schools’ efforts in addressing learners’ barriers to learning.
